# Physeal sparing technique reduces femoral growth disturbance in pediatric anterior cruciate ligament reconstruction patients

**DOI:** 10.1002/ksa.12763

**Published:** 2025-07-02

**Authors:** Torsten Grønbech Nielsen, Jannie Bøge Steinmeier Larsen, Mette Mølby Nielsen, Michel Bach Hellfritzsch, Peter Ziegler Faunø, Martin Lind

**Affiliations:** ^1^ Sports Traumatology, Orthopedic Department Aarhus University Hospital Aarhus Denmark; ^2^ Department of Physiotherapy and Occupational Therapy Aarhus University Hospital Aarhus Denmark; ^3^ Department of Radiology Aarhus University Hospital Aarhus Denmark; ^4^ Department of Clinical Biochemistry Aarhus University Hospital Aarhus Denmark

**Keywords:** anterior cruciate ligaments, children, growth plate, knee injury, treatment outcome

## Abstract

**Purpose:**

The purpose of this study was to compare the difference in limb length and angular deformity between two cohorts of patients who have underwent anterior cruciate ligament (ACL)‐reconstruction using a femoral non‐physeal sparing technique (N‐PS) and a physeal sparing technique (PS). It was hypothesised that N‐PS would result in less discrepancy in limb length and knee angles than PS.

**Methods:**

This is a comparative cohort study of 113 patients: 33 in N‐PS who underwent ACL reconstruction between 2001 and 2010, and 80 in PS who underwent ACL reconstruction between 2013 and 2019. Radiographic evaluation, knee stability measurements and patient‐reported outcomes (PROMS) were completed at the two‐year minimum follow‐up examination. Student's t‐test, Wilcoxon signed‐rank sum test and chi‐squared test were used to analyse the data.

**Results:**

At time of surgery, the patients were 11.7 (standard deviation [SD]: 1.4) and 14.0 (SD: 1.3) years old in N‐PS AND PS, respectively. N‐PS showed a statistically significant difference in femoral length of 3.5 mm (95% confidence interval [CI]: 1.1–5.9) compared to the non‐operated side, with no effect on femoral length in PS of 1.0 mm (95%CI: 0.0–1.9).Only a minor impact on tibial angulation (*p* = 0.07) was observed between the techniques. Sensitivity analysis showed that patients younger than 12.5 years at time of surgery were five times more likely to have a total length difference of more than 10 mm in N‐PS than in PS (*p* = 0.05). Statistically significant differences (*p* = 0.02) were observed in knee laxity between the N‐PS 1.5 mm (95%CI: 1.0–1.2) and PS 2.4 mm (95%CI: 1.9–2.8).

**Conclusions:**

A femoral physeal sparing technique for paediatric ACL‐reconstruction reduced femoral limb length growth disturbance compared with non‐physeal sparing technique. Knee laxity was affected and PROMS were unaffected by the type of technique.

**Level of Evidence:**

Level III.

**Abbreviations**
ACL: anterior cruciate ligament
ADL: activity of daily living
AUH: Aarhus University Hospital
CI: confidence intervals
KOOS: knee injury and osteoarthritis outcome score
mm: millimetres
n: numbers
PROMS: patient‐reported outcomes
QOL: quality of life
SD: standard deviation
Tegner: tegner activity scale

## INTRODUCTION

Approximately 2500 anterior cruciate ligament (ACL) reconstructions are performed each year in Denmark [21], of which approximately 6% are performed in children under the age of 15 [22].

Several studies have shown that the incidence of ACL injuries in children and adolescents has increased significantly in recent years [30, 35].

The optimal treatment strategy for ACL injuries in children and adolescents is still unclear and may consist of either a non‐operative management with rehabilitation focused on increasing muscle strength, or operative management with ACL reconstruction [14]. When the treatment strategy is rehabilitation without surgery, there is increased knee instability, increased risk of meniscal injury and reduced ability to return to sport compared to patients who undergo surgery [14].

A study by Moksnes et al. found that approximately 22% of children with ACL injuries who received rehabilitation rather than surgery, underwent surgery within the first 2 years of sustaining the knee injury, although the patients were not required to return to pivoting sports [26]. Therefore, ACL reconstruction is highly relevant for children with ACL injuries.

ACL reconstruction in children can potentially damage the growth zones around the knee, affecting limb length growth and knee angles. Based on the existing literature, it is unclear whether ACL reconstruction in children can affect limb length and knee angles. Some studies have found no impact on limb length and knee angles [1, 3, 18], while others have found differences of up to 24% [6, 19, 23]. The measurement methods used in the studies vary, making it difficult to compare results between studies. The reason for a possible difference in limb length and knee angles after ACL reconstruction is trauma to the femoral and tibial growth plates due to tunnel drilling [30]. Therefore, consideration should be given to using a surgical technique that protects the growth plates as much as possible, while still providing an optimal ACL reconstruction technique.

The change in surgical technique in Aarhus University Hospital (AUH) provided an opportunity to compare the outcome and impact on growth disturbance of the two different surgical techniques.

The purpose of this study was to compare the difference in limb length and knee angles between two groups of patients who underwent ACL reconstruction as children using a femoral physeal sparing technique and a non‐physeal sparing technique at the AUH. It was hypothesised that patients undergoing ACL reconstruction using the physeal‐sparing technique would have less discrepancy in limb length and knee angles than those receiving the non‐physeal‐sparing technique. Second, to compare measurements of knee stability and patient‐reported questionnaires (PROMS) of knee function and quality of life between the two groups.

## MATERIALS AND METHODS

This is a comparative cohort study with data from two cohorts consisting of skeletally immature patients who underwent ACL reconstruction using two different surgical techniques between 2001–2010 and 2013–2019, respectively. The study was conducted in accordance with the STROBE guidelines to ensure transparency and systematic reporting of methods and results [32]. In 2011, the surgical technique at AUH was changed from a femoral non‐physeal sparing ACL reconstruction technique to a femoral physeal sparing technique. Clinical outcomes with both techniques have been presented individually in previous studies, but the results have not been compared [6, 7]. Inclusion criteria for both cohorts were preoperative open physis in the distal femur and proximal tibia prior to surgery, desire to return to pivoting sport and/or failure of non‐operative treatment. Exclusion criteria were multiligament injuries, revision ACL reconstruction and children with open physis in the tibia and femur at follow‐up. All ACL reconstructions were performed at the AUH by a single surgeon.

### Surgical techniques and rehabilitation

**Figure 1:**
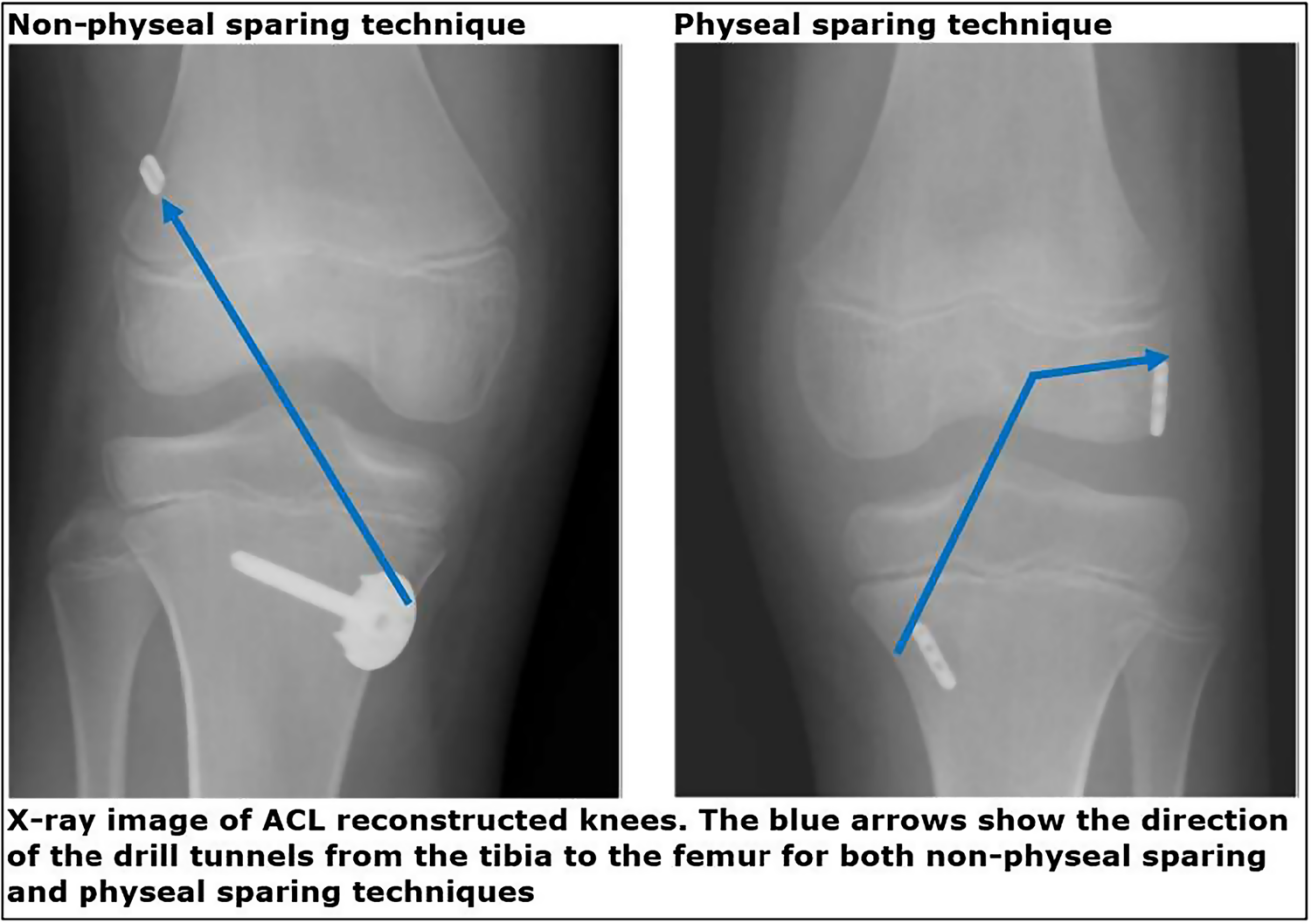
Surgery techniques. ACL, anterior cruciate ligament.

A transphyseal drilled ACL reconstruction was performed using a doubled semitendinousus and gracilis autograft. An Endobutton CL loop implant (Smith & Nephew, Andover, USA) was used for femoral fixation and a bicortical screw and washer (Arthrex, Naples, USA) for tibial fixation. Both the femoral and tibial growth plates were crossed. The femoral tunnel was drilled through an anteromedial portal [6] (Figure 1).

For ACL reconstruction using the femoral physeal sparing technique, an ipsilateral harvested quadruple semitendinosus autograft was used and secured at both ends with extracortical fixation (Rigidloop, DepuySynthes, Warsaw, USA). The tibial tunnel was placed transphyseal, while the femoral tunnel was placed below the physis using fluoroscopically guided retrodrilling. The diameter of the drilled tunnels was adjusted according to the size of the graft [7] (Figure 1).

Patients in both groups followed the same rehabilitation programme. They were allowed to full weight‐bearing post‐operatively. No bracing was used. All patients received physiotherapist‐assisted rehabilitation within the first year. Return to non‐weight‐bearing sports was allowed 6 months postoperatively, and full return to contact sports was not recommended within the first 12 months after ACL reconstruction [6, 7].

### Primary and secondary outcomes

At final follow‐up, radiological assessment, clinical outcomes and PROMS were assessed and compared with preoperative status and between the two cohorts.

### Primary outcome

The primary outcome of this study was the radiological assessment at final follow‐up, including full limb radiographs to assess limb length discrepancies and coronal malalignment. Growth disturbances were assessed at skeletal maturity by high‐precision axial and coronal radiographic analysis, comparing the operated and non‐operated lower limbs. All radiological examinations were performed by an experienced radiographer, and subsequent measurements on the radiological images were evaluated by both an experienced radiographer and a radiologist. Neither the radiographer nor the radiologist were blinded to the surgical technique during the radiological assessment.

The multiDiagnost Eleva 3D‐RX system (Phillips Medical Systems) was used for the non‐physeal sparing technique cohort [6]. The Adora DRFi system (NRT X‐RAY A/S, Hasselager, Denmark) was used for the physeal sparing technique cohort [7]. Both have an optional lower limb geometry measurement application.

Radiographs for limb measurements were obtained using the standard acquisition protocol, with patients standing upright, with their backs against the vertical table top, and full weight bearing on the limb being examined. A detailed description can be found in the previous publication [7].

An axial limb length discrepancy of more than 10 mm and a side‐to‐side angle difference of more than 5° in the coronal plane were defined as clinically significant [2, 7, 33].

### Sensitivity analysis and secondary outcomes

As the age difference between the cohorts varied, a sensitivity analysis was performed comparing children younger than 12.5 years in the two groups [25].

Secondary outcomes were objective outcomes including sagittal knee laxity and rotational knee laxity and PROMS including the Knee Injury and Osteoarthritis Outcome Score (KOOS) and the Tegner Activity Scale (Tegner).

Instrumented sagittal knee laxity was a side‐to‐side difference measured with a KT‐1000 or a Rolimeter and differences were reported in millimetres [8].

Rotational laxity was assessed using the pivot shift test. A negative pivot shift was scored as 0, whereas a score of 1–3 was considered a positive pivot shift [12].

The KOOS score was originally developed for patients with knee osteoarthritis [29]. It has five domains; pain, symptoms, activity of daily living (ADL), sport and quality of life (QOL), with sport and QOL showing good validity in ACL reconstructed patients [13]. The total score for each domain ranges from 0 to 100, with 0 representing extreme knee problems and 100 representing no knee problems.

Tegner was a single‐item activity scale using an 11‐point Likert scale ranging from 0 to 10, with 0 representing maximum functional disability and 10 representing elite level of sport [31].

### Data evaluation and statistical analysis

All statistical analyses were performed with Stata 18.0 (StataCorp. 2021. Stata statistical software: Release 18. College Station, TX: StataCorp LLC).

Continuous data that was normally distributed, such as age, follow‐up time, knee laxity, limb length and knee angular differences, were presented as means with standard deviations (SD) or 95% confidence intervals (95% CI) and analysed using a Student's t‐test for comparison. Non‐normally distributed data, such as KOOS subscores and the Tegner score, were presented as medians with interquartile ranges and analysed using the Wilcoxon signed‐rank test.

Binomial and categorical variables such as sex, pivot shift, pivoting sport, meniscal and cartilage damage, tibial eminence fracture, and limb differences were presented as numbers (*n* (%)) and compared using the chi‐squared or Fisher's exact test (frequency < 5). *p* < 0.05 was considered statistically significant.

## RESULTS

Between 2001 and 2010, 39 patients with primary isolated ACL lesions underwent ACL reconstruction at the AUH using the non‐physeal sparing technique. Six patients declined to participate, leaving 33 patients for follow‐up. Patients who underwent revision ACL surgery were not included in this cohort, as ACL revision was an exclusion criterion in this early cohort.

**Figure 2:**
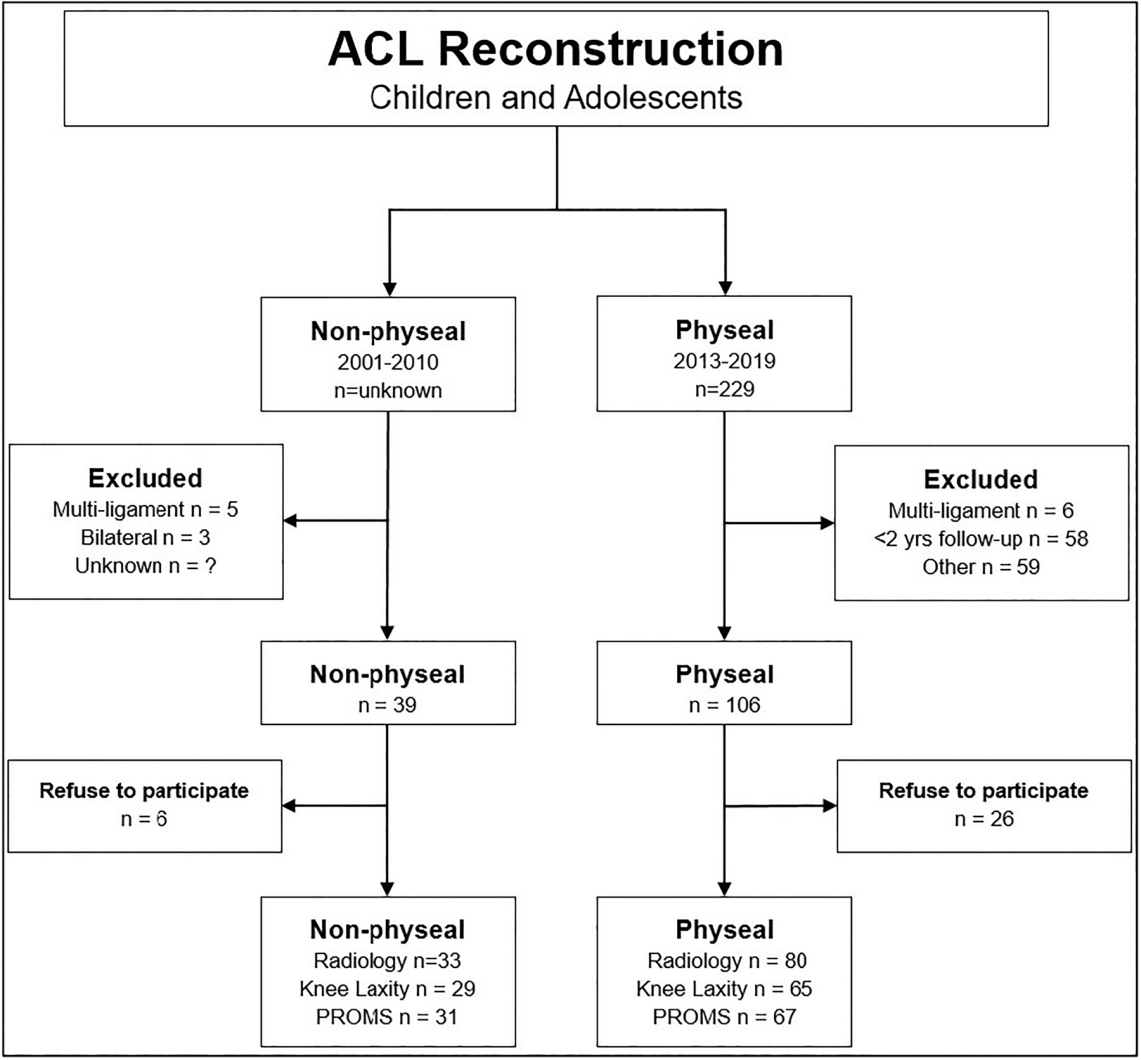
Flow‐chart algorithm.

Between 2013 and 2019, 229 ACL patients underwent surgery at the AUH using the physeal sparing technique. Of these, 106 patients met the inclusion and exclusion criteria. Twenty‐six patients refused to participate in the study. Of the 80 patients, the secondary results of 13 patients could not be used in the analysis of knee laxity and PROMS, because the patients underwent additional ACL surgery after the primary ACL surgery (Figure 2).

The comparative cohort study included a total of 113 adolescents who underwent ACL reconstruction as children, 67 boys (59%) and 46 girls (41%). Differences in age at surgery, age at follow‐up, follow‐up time and in the mechanism of injury were observed between the two cohorts with the physeal sparing technique cohort being older and more likely to be injured in pivoting sport than the non‐physeal sparing technique cohort (Table 1).

**Table 1:** Baseline characteristics.

	Non‐physeal sparing technique (*n* = 33)	Physeal sparing technique (*n* = 80)	*p* value
Age at surgery, mean ± SD	11.7 ± 1.4	14.0 ± 1.3	<0.001
Age at follow‐up, mean ± SD	18.3 ± 3.8	19.5 ± 1.4	0.02
Follow‐up time, months mean ± SD	78.6 ± 41.4	61.9 ± 13.0	0.001
Sex, female/male *n* (%)	11/22 (33/67)	35/45 (44/56)	0.31
Knee laxity, mm mean (95%CI)	5.1 (3.8–6.3)	5.1 (4.8–5.3)	0.95
Pivot shift test, grade = 0 *n* (%)	2 (6.1)	0 (0.0)	
Pivoting sport leading to injury, *n* (%)	9 (37.5)	58 (78.4)	0.001
Meniscal damage, *n* (%)	6 (18.2)	18 (22.5)	0.61
Cartilage damage, *n* (%)	1 (3.0)	3 (3.8)	0.85
Previous surgery			
− Tibial eminence fracture, *n* (%)	1 (3.0)	2 (2.5)	0.27
− Other, *n* (%)	4 (12.1)	6 (7.5)	
− None, *n* (%)	28 (84.9)	72 (90)	
KOOS	(*n* = 12)	(*n* = 31)	
− Symptoms, median (quartiles)	66 (59;73)	82 (75;86)	0.01
− Pain, median (quartiles)	64 (43;78)	86 (64;92)	0.01
− ADL, median (quartiles)	69 (53;86)	94 (82;97)	0.003
− Sport, median (quartiles)	43 (8;53)	60 (25;75)	0.16
− QOL, median (quartiles)	38 (31;47)	38 (25;44)	0.90
Tegner activity score	(*n* = 39)	(*n* = 31)	
− Before injury, median (quartiles)	7 (6;7)	7 (6;9)	0.16
− Before surgery, median (quartiles)	1.5 (1;4)	3 (1;4)	0.21

Abbreviations: ADL, Activity of Daily Living; CI, Confidence Interval; KOOS, Knee Injury and Osteoarthritis Outcome Score; *n*, numbers; mm, millimetres; QOL, Quality of Life; SD, standard deviation.

In the non‐physeal sparing cohort, there was a statistically significant difference in femur length (3.5 mm [1.1; 5.9]) and total limb length difference (3.6 mm [0.7; 6.6]). There was also a statistically significant difference in femoral (−1.7 degrees [−2.5; −0.9]) and in tibial (1.0 degrees [0.2; 1.86]) angles. However, the mean differences in limb length and knee angle were below clinically relevant thresholds.

In the physeal sparing cohort, there was a statistically significant difference in femoral angle (−1.7 degrees [−2.4; −1.0]) which was not considered clinically relevant. No differences were found for limb length or tibial knee angle (Table 2).

**Table 2:** Primary outcome.

	Non‐physeal sparing technique (*n* = 33)	Physeal sparing technique (*n* = 80)	*p* value (between techniques)
Limb length differences			
− Femur (mm), mean (95%CI)	3.5 (1.1–5.9)*	1.0 (0.0–1.9)	0.02
− Tibia (mm), mean (95%CI)	0.9 (−0.6 to 2,3)	−0.7 (−1.5 to 0.2)	0.05
− Total (mm), mean (95%CI)	3.6 (0.7–6.6)*	0.1 (−1.4 to 1.5)	0.02
Knee angular differences			
− Femur (degrees), mean (95%CI)	−1.7 (−2.5 to −0.9)*	−1.7 (−2.4 to −1.0)*	0.91
− Tibia (degrees), mean (95%CI)	1.0 (0.2–1.8)*	0.1 (−0.4 to 0.6)	0.07

Abbreviations: CI, confidence interval; mm, milimetres; *n*, numbers.

* Statistic significant difference (Student's t‐test) between operated and non‐operated leg within the two techniques.

A statistically significant difference in the proportion of patients with a difference in total limb length greater than 10 mm was found between the two cohorts (27% vs. 11% for non‐physeal sparing and physeal sparing, respectively; *p* = 0.01). No statistical difference in limb length was found between patients with a difference of >10 mm for non‐physeal sparing (14.9 mm, 95%CI:11.7–18.1) and physeal sparing (14.0 mm, 95%CI: 12.6–15.4). Of the 13 ACL revision patients in the physeal sparing cohort, one patient (8%) had a limb length difference of >10 mm. No differences were observed for femoral or tibial angle differences (Table 3).

**Table 3:** Clinically significant limb difference.

	Non‐physeal sparing technique (*n* = 33)	Physeal sparing technique (*n* = 80)	*p* value
TOTAL ‐ Limb length differences			
>10 mm, *n* (%)	9 (27)	9 (11)	0.03
<10 mm, *n* (%)	24 (73)	71 (89)	
FEMUR ‐ Knee angular differences			
>5 degrees, *n* (%)	5 (15)	12 (15)	0.98
<5 degrees, *n* (%)	28 (85)	68 (85)	
TIBIA ‐ Knee angular differences			
>5 degrees, *n* (%)	1 (3)	5 (6)	0.49
<5 degrees, *n* (%)	32 (97)	75 (94)	

Abbreviations: mm, millimetre; *n*, numbers.

Sensitivity analysis showed that patients younger than 12.5 years of age at the time of surgery had a five‐fold higher risk ratio (40% vs. 8%) of having a total length difference >10 mm when the surgical technique was non‐physeal sparing compared with physeal sparing (Table 4). In patients older than 12.5 years of age, there was no difference between groups.

**Table 4:** Clinically significant limb difference–Age stratification.

	Age <12.5 years (*n* = 32)		*p* value	Age >12.5 years (*n* = 81)		*p* value
	Non‐physeal sparing technique (*n* = 20)	Physeal sparing technique (*n* = 12)		Non‐physeal sparing technique (*n* = 13)	Physeal sparing technique (*n* = 68)	
Total ‐ limb length differences						
>10 mm, *n* (%)	8 (40)	1 (8)	0.05	1 (8)	8 (11)	0.67
<10 mm, *n* (%)	12 (60)	11 (92)		12 (92)	60 (89)	

Abbreviations: mm, millimetre; *n*, numbers.

A statistical significant difference in sagittal knee laxity was found between the non‐physeal sparing technique (1.5 mm [95%CI: 1.0–2.1]) and the physeal sparing technique (2.4 mm [1.9–2.8]). No differences were found in terms of rotational instability, with 79% and 66% of patients in the non‐physeal sparing technique and the physeal sparing technique, respectively, having a pivot shift grade = 0.

The results for patient‐reported functional level, as measured by the KOOS and Tegner questionnaires after surgery, did not differ between the two surgical techniques (Table 5).

**Table 5:** Secondary outcomes.

	Non‐physeal sparing technique	Physeal sparing technique	*p* value
Clinical outcomes	(*n* = 29)	(n = 65)	
Sagittal knee laxity, mm mean (95%CI)	1.5 (1.0–2.1)	2.4 (1.9–2.8)	0.02
Pivot shift test = grade 0, *n* (%)	23 (79)	43 (66)	0.15
KOOS	(*n* = 31)	(*n* = 67)	
− Symptoms, median (quartiles)	79 (71;89)	82 (71;93)	0.56
− Pain, median (quartiles)	89 (83;97)	94 (83;100)	0.43
− ADL, median (quartiles)	96 (88;100)	99 (91;100)	0.35
− Sport, median (quartiles)	80 (60;90)	85 (65;95)	0.37
− QOL, median (quartiles)	75 (63;88)	81 (50;94)	0.69
Tegner activity score, median (quartiles)	7 (5;7)	5 (4;7)	0.06

Abbreviations: ADL, activity of daily living; CI, confidence interval; KOOS, knee injury and osteoarthritis outcome score; *n*, numbers; mm, millimetres; QOL, quality of life; SD, standard deviation.

## DISCUSSION

The primary finding of this study was that ACL reconstruction in skeletally immature patients using a femoral physeal sparing technique reduced the growth disturbance in terms of limb length discrepancy compared to a non‐physeal sparing technique.

This is the first comparative study to report this finding. Only one systematic review by Pierce et al. found limited differences between non‐physeal sparing and physeal sparing techniques in terms of growth disturbances [28]. The review found 0.8% of limb length discrepancy (>10 mm) and angular deformity in 0.6% of 491 patients in 18 studies of the non‐physeal sparing technique. For the physeal sparing technique, limb length discrepancy (>10 mm) was found in 1.3% of patients and angular deformity in 0.0% of 162 patients in six studies. In the present study, limb length discrepancy >10 mm was found in 27% of non‐physeal sparing ACL patients and 11% of patients with a physeal sparing technique. No difference was found for angular deformity, as both groups had 15% of patients with more than 5 degrees of angular deformity. None of the patients in this study had symptoms from the deformities or required surgical intervention. Thus, the present study found a preventive effect of the physeal sparing technique on growth disturbance, which differs from the results of the systematic review. The different results could be explained by the many different measurement techniques used in the typically small cohort studies included in the systematic review, compared with the high‐precision radiographic methods used in the present study, which also had a relatively large number of patients (113 patients).There are conflicting data regarding the incidence of growth disturbances following ACL reconstruction in skeletally immature patients. The rate of growth complications has been reported to range from 0% to 24% depending on the surgical technique used [5, 7, 16, 17]. In a meta‐analysis of 1321 patients by Wong et al., 4.4% of the patients developed growth disturbances, of which one third required corrective surgery [36]. Another systematic review by Collins et al. looked at what growth disturbances were associated with non‐physeal sparing and physeal sparing techniques in 21 case series. The review found an overall incidence of growth disturbance of 13% and that the femoral physeal sparing technique was associated with varus deformity and the tibial non‐physeal sparing technique was associated with limb shortening [2].

In the present study, 27% and 11% of patients in the two groups, respectively, had a length growth deformity of more than 10 mm. This is in contrast to what was reported in the systematic reviews, where the incidence of growth disturbances ranged from 5%–13% [2, 16, 34]. However, at least one of the reviews suggests a potential serious underreporting bias in previous studies on this topic [2]. Limb length inequality is relatively common in the adult population [11]. It is therefore difficult to estimate how much of the found growth deformity found is within the normal anatomical variation. Small differences in limb length have not been found to cause gait abnormalities [9]. It is only when the limb length difference exceeds 2 cm, that significant gait asymmetry is seen [15].

It has been suggested that a potential physis injury may occur during ACL reconstruction even without transphyseal drilling as a result of thermal influence due to the proximity of the growth plate to the physeal sparing tunnel drilling [20], and this phenomenon has also been described in an animal laboratory study [24]. This study found no significant differences in limb length or tibial angle in the physeal‐sparing cohort. However, difference in femoral angle was observed. This difference was below clinical relevance.

A statistically significant difference of 3.6 mm (95%CI:0.7–6.6, *p* = 0.02) in total limb length was found between the legs in the non‐physeal sparing group. However, although this difference was significant, it was clinically irrelevant. As Vogt et al. stated, a discrepancy of less than 10 mm is within the normal range [33].

We did not find any clinically relevant growth disturbance in the coronal plane with either of the two ACL reconstruction methods studied. A previous study described the occurrence of tibial angular deformity after paediatric ACL reconstruction using trans‐physeal drilling [6]. In this previous study, a trans‐physeal tibial tunnel was drilled. The tibial tunnel was oblique and therefore crossed the physis at a more peripheral point. The more oblique tibial tunnel also creates a larger oval‐shaped and more anteriorly peripheral placed growth plate injury. The present physeal sparing surgical techniques, used a tibial retro‐drill that could be placed independently of the intended femoral tunnel placement and with a more vertical and centrally placed tunnel tibial tunnel. This may explain the lack of tibial malalignment findings in the present study.

Surprisingly, the sensitivity analyses showed that 40% of patients younger than 12.5 years of age with a non‐physeal sparing technique had a limb length discrepancy. This contrasts with the findings of Demange and Camanho, who found no limb length discrepancy in their 12 ACL reconstructed patients under the age of 12 years [4]. A systematic review of 78 studies found a limb length discrepancy in 27 of 2693 patients, which was also lower than the results of this study. None of the studies included in the review reported comparable limb length discrepancies [27]. The results of the studies were also lower than the physeal sparing technique of 8% in this study. Using the present physeal sparing technique in ACL patients resulted in a five‐fold reduction in the risk of limb length discrepancy in patients younger than 12.5 years. Children of a younger age have a higher growth potential and therefore a sensitivity analysis may show differences between age groups. The risk of limb length discrepancy in patients older than 12.5 years of age was not affected by the surgical technique.

An increased sagittal side‐to‐side difference of 2.3 mm at two years was found for the physeal sparing technique compared to a non‐physeal sparing technique, which was 1.7 mm at two years. We found no relevant explanation for this finding of increased knee laxity. It is debatable whether a difference of 0.6 mm in sagittal knee laxity is clinically relevant.

Patients in both surgical technique groups had a good subjective clinical outcome in terms of knee function, with KOOS scores close to normal and no difference between groups. Compared to a recent study evaluating paediatric ACL reconstruction by Hansson et al. [10], the subjective outcome in the present study was comparable to the postoperative KOOS scores.

The clinical significance of the present study is the finding that femoral limb length disturbance can be reduced by using a femoral physeal sparing technique. Therefore, the study suggests that ACL reconstruction in immature patients can be performed without growth disturbances and that the femoral physeal sparing technique can therefore be recommended for immature patients of all ages with ACL injuries undergoing reconstructive surgery. This study did not evaluate growth changes in the sagittal plane. However, although a difference in limb length disturbance was found between the two techniques, no difference in PROMs was observed, indicating that the difference may not be clinically relevant.

A limitation of the present study was the difference in age between the two groups. As younger patients have a higher growth potential this study included a sensitive analyses to make the groups more comparable. A preoperative radiological bone age assessment would have provided a more nuanced estimate of the remaining growth potential for each patient.

Another limitation was the absence of a power analysis investigating limb differences. As no previous studies have compared these two surgical techniques and studies investigating limb differences were conducted on small sample sizes, a power analysis supported by the literature was difficult to conduct. Consequently, the study may be underpowered for binomial outcomes.

In addition, the study design was with a historical control group comparison rather than a randomised design, which would have improved the scientific value of the study results.

## CONCLUSION

A femoral physeal sparing technique for paediatric ACL reconstruction reduced femoral limb length growth disturbance compared with a non‐physeal sparing ACL reconstruction technique. Coronal plane growth disturbance was unaffected. Knee laxity and PROMS were unaffected by the type of ACL reconstruction technique.

Although a difference in limb length disturbance was found between the two techniques for ACL reconstruction in ACL‐injured patients with open growth plates, no difference in PROMs was observed, indicating that the difference may not be clinically relevant.

## AUTHOR CONTRIBUTIONS

All authors contributed to the study conception and design. Data analysis were performed by Torsten Grønbech Nielsen. Analysis and interpretation of the results was done by both authors. Martin Lind was a major contributor in writing the manuscript. Both authors read and approved the final manuscript.

## CONFLICT OF INTEREST STATEMENT

The authors declare no conflicts of interest.

## ETHICS STATEMENT

Please include the name of the institutional review board (IRB) and the approval number. If not applicable, please state so. The study was conducted in accordance with the Helsinki Declaration and approved by the local ethics committee (1‐10‐72‐246‐20). The study was registered in the region's internal database of research projects. All patients received written and verbal information about the study and participation was voluntary. All patient completed informed consent form.

## Data Availability

Data are available on request.
